# Statistical independence for the evaluation of classifier-based diagnosis

**DOI:** 10.1007/s40708-014-0007-6

**Published:** 2014-12-11

**Authors:** Emanuele Olivetti, Susanne Greiner, Paolo Avesani

**Affiliations:** 1 NeuroInformatics Laboratory (NILab), Fondazione Bruno Kessler, Trento, Italy; 2 Centro Interdipartimentale Mente e Cervello (CIMeC), Università di Trento, Trento, Italy

## Abstract

Machine learning techniques are increasingly adopted in computer-aided diagnosis. Evaluation methods for classification results that are based on the study of one or more metrics can be unable to distinguish between cases in which the classifier is discriminating the classes from cases in which it is not. In the binary setting, such circumstances can be encountered when data are unbalanced with respect to the diagnostic groups. Having more healthy controls than pathological subjects, datasets meant for diagnosis frequently show a certain degree of unbalancedness. In this work, we propose to recast the evaluation of classification results as a test of statistical independence between the predicted and the actual diagnostic groups. We address the problem within the Bayesian hypothesis testing framework. Different from the standard metrics, the proposed method is able to handle unbalanced data and takes into account the size of the available data. We show experimental evidence of the efficacy of the approach both on simulated data and on real data about the diagnosis of the Attention Deficit Hyperactivity Disorder (ADHD).

## Introduction

Classification-based machine learning techniques are increasingly adopted in computer-aided diagnosis because they have limited need for a pathophysiological model of the disease under investigation. The efficacy of such *model-free* approaches depends on many factors, like the size of the available training sample. A bigger sample size allows for the training of a more robust classifier and might improve the prediction accuracy (PA) on the test set. However, even an enormous amount of data does not guarantee the correct diagnosis of a disease via classifier.

Once a suitable classification algorithm has been trained, its efficacy has to be assessed by predicting the diagnostic groups of subjects in a test set and comparing them against the true values. It is common practice to calculate one or more metrics such as the PA, F1-Score, Matthews Correlation Coefficient, $\kappa$-statistic [6, 11, 12] or AUC/ROC [3, 13] to decide whether the classifier is able to discriminate between healthy controls and one or more stages/types of the pathology of interest. Each of those metrics has different strengths and drawbacks. For example, PA is not able to properly handle datasets where the number of available examples per class is not equal, a setting referred to as *unbalanced*. More importantly, common metrics for evaluating classifiers do not depend on the actual test set size, i.e. they do not measure the amount of evidence the results of prediction provide.

**Fig. 1:**
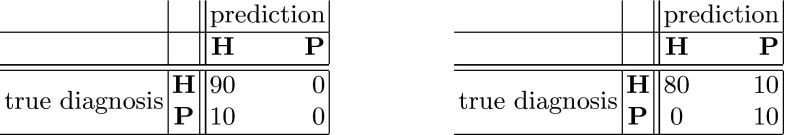
Two simulated examples of confusion matrices, true class labels on the rows and predicted class labels on the columns. Both confusion matrices have the same prediction accuracy (PA), i.e. $PA = 1 - \hat{\epsilon } = \frac{0+90}{100} = \frac{80+10}{100} = 90\,\%$. Nevertheless, in the first case, there is no evidence that the classifier is able to discriminate **H** from **P**, while in the second one there is.

Through an example, we illustrate how a metric, in this case the PA, can be influenced by the characteristics of the dataset, e.g. unbalancedness. Given a test set of 100 subjects where 90 are healthy controls (**H**) and 10 are patients (**P**), a classifier that obtains 90 % of PA could be either highly accurate in discriminating the two classes, and therefore diagnosing the disease, or not able at all. These two extreme cases are illustrated in Fig. 1 by means of their confusion matrices.A confusion matrix reports the joint results of the predicted and the true diagnostic groups. The table on the left shows a classifier that always predicts the most frequent diagnostic group, i.e class **H**, which is a typical issue of classifiers that are not able to discriminate the two classes. The table on the right shows a classifier that correctly discriminates the two diagnostic groups and incurs in only 10 errors over 90 examples in the most frequent class **H**.

The confusion matrix is a convenient way to represent results of a classifier because all the metrics used to evaluate classifiers can be computed from it. In the same way, the method that we propose in this work is based on the analysis of the confusion matrix. Specifically, we propose to quantify the evidence between two alternative hypotheses about the underlying generation mechanism of the observed confusion matrix. The first hypothesis is that the predicted class labels are statistically *independent* from the true class labels. This is the case were the classifier is not able to discriminate the classes. The second hypothesis is that the predicted class labels are statistically *dependent* on the true class labels. In this case, the classifier predicted according to the true class labels. The degree of evidence in favour of one hypothesis or of the other is the measure that we propose for evaluating the classifier.

In order to implement the proposed method, we draw from the statistics literature and adopt a recent Bayesian test of independence for contingency tables [2], which was proposed in a context different from that of classification. The proposed method is able to handle imbalancedness, takes the sample size of the test set into account and provides the correct answer in cases in which standard metrics are misleading. Furthermore, this kind of approach can be extended to the multi-class setting, while traditional evaluation methods are often tailored and limited to the binary setting. We defer the presentation of the multi-class case to future work.

In the following, we describe the standard classification task for diagnosis in medical image analysis and define where the problem concerning result evaluation lies. Subsequently, we introduce the Bayesian test of independence and show its efficacy on a simulated toy example and on real data concerning the computer-aided diagnosis of the attention deficit hyperactivity disorder (ADHD).

## Methods

The first part of this section formally defines the notation and framework of classification-based diagnosis. The second part introduces the Bayesian hypothesis testing framework and the proposed solution to the problem of evaluating the classification result.

### Classification-based diagnosis

Let ${\mathcal{X}}={\mathbb{R}}^d$ be the multidimensional feature space under investigation, e.g. medical image data, and let ${\mathcal{Y}}=\{1,\ldots ,c\}$ be the set of classes that represents the possible values of the variable of interest.

Each training example is then a vector ${\mathbf{X}} \in {\mathcal{X}}$, e.g. the data from one subject of the study, with class label $Y \in {\mathcal{Y}}$, e.g. the subject’s pathology. Let $P_{XY}$ be the unknown joint distribution over ${\mathcal{X}} \times {\mathcal{Y}}$. We are given a previously trained classifier $f:{\mathcal{X}} \rightarrow {\mathcal{Y}}$ that predicts the variable of interest given the data about which the performance is to be determined. We call $\epsilon = E_{XY}[f({\mathbf{X}}) \ne Y]$ the generalization error of $f$.

In practical cases, the test set is of finite size $m$, therefore we do not know the actual joint distribution $P_{XY}$ and $\epsilon$ can only be estimated. Let $S=\{(x_1,y_1),\ldots ,(x_m,y_m)\}$ be the test set which is assumed to be an i.i.d. set of observations, i.e. examples, drawn from $P_{XY}$. The standard estimator of $\epsilon$ is $\hat{\epsilon } = \frac{e}{m},$ where $e$ is the total number of misclassified examples.

The set of true class labels and predicted class labels can be summarized by the confusion matrix $\varvec{Z}$, which is a contingency table (see Fig. 1) that reports the number of occurrences $z_{ij}$ of each possible pair of outcomes. The sum $\sum {z_{ij}} = m$ equals the test set size and the diagonal contains all correctly classified examples $\sum _{i}{z_{ii}} = m-e$. The estimated PA is defined as $PA = \frac{m-e}{m} = 1-\hat{\epsilon }$.

For the binary case, the two values on the diagonal of $\varvec{Z}$ are defined as true positive ($TP$) and true negative ($TN$), respectively, and Type I error as false positive ($FP$) as well as Type II error as false negative ($FN$). The sensitivity or true positive rate $TPR$, the false positive rate ($FPR$) and specificity ($SPC$) are defined as

1 $$TPR = \frac{TP}{TP+FN} \quad \quad FPR = \frac{FP}{FP+TN} \quad \quad SPC = \frac{TN}{FP+TN}$$

and the Matthews correlation coefficient ($MCC$), the $F1$-score [11, 12] and the $\kappa$-Statistic [6] as

2 $$\begin{aligned} MCC&= \frac{ TP \times TN - FP \times FN }{\sqrt{ (TP + FP) ( TP + FN ) ( TN + FP ) ( TN + FN )}} \end{aligned}$$

3 $$\begin{aligned} F1&= 2 \frac{TP}{2TP + FP + FN} \end{aligned}$$

4 $$\begin{aligned} \kappa&= \frac{PA - rPA}{1 - rPA} \end{aligned},$$

where

$$\begin{aligned} rPA &= \frac{(TN + FP)(TN+ FN)+(FN+TP)(FP+TP)}{m^2}. \end{aligned}$$

The receiver operating characteristic (ROC) analysis is the study of the curve of $FPR$ versus $TPR$ while a sensitivity or threshold parameter is varied [3]. In our case, classification has already been performed, meaning that there is no such parameter variation and the ratio between both rates is fixed. Nonetheless, the intercept of the ROC point with the line at 90 degrees to the no-discrimination line, also known as Youden’s $J$ statistic [13], can be used as a measure for the evaluation of performance:

5 $$J = TPR + SPC - 1. $$

### Evaluation by the Bayesian test of independence

As noted in [8], when data are unbalanced with respect to the class-label distribution, the PA (or the misclassification error rate) of a classifier can be a misleading statistic to assess whether the classifier actually discriminated the classes or not. An alternative solution to the issue of evaluating classifiers through the error rate/accuracy is testing the full confusion matrix.

The literature answering the question “did the classifier learn to discriminate the classes?” was recently reviewed in [8], and a novel approach based on the analysis of the statistical independence between predicted and true class labels was proposed based on the work of [2]. In this work we adopt a similar approach that we summarise here. The intuitive idea is that, following the definition of statistical independence between random variables, in the case of a classifier predicting at random, the predicted class labels are statistically independent of the true class labels. Conversely, the more the predictions match the true class labels, the stronger is the statistical dependence between them. We propose the use of the Bayesian test of independence in contingency tables described in [2] to compute the ratio of the posterior probabilities of the following two hypotheses:

- ${\bf{H_0}}$: the predictions are statistically *independent* of the true class labels.
- ${\bf{H_1}}$: the predictions are statistically *dependent* on the true class labels.

According to the Bayesian hypothesis testing framework [5], the comparison of two hypotheses can be quantified by the ratio of their posterior probabilities. That ratio can be rewritten as

6 $$\frac{P(H_1|\varvec{Z})}{P(H_0|\varvec{Z})} = \frac{P(H_1)}{P(H_0)}\frac{P(\varvec{Z}|H_1)}{P(\varvec{Z}|H_0)} = \frac{P(H_1)}{P(H_0)} B_{10},$$

where $B_{10}$ is called *Bayes factor* and measures the evidence of the data in favour of $H_1$ with respect to $H_0$. When $B_{10} \ggg 1$ the evidence is in favour of $H_1$ against $H_0$. Guidelines for the interpretation of the strength of evidence are presented in [5] and reported here in Table 1.

**Table 1:** Guidelines for the interpretation of the logarithm of the Bayes factor $log(B_{10})$ in terms of the strength of evidence in favour of $H_1$ and against $H_0$, from [5]

$log(B_{10})$	<0	0 to 1	1 to 3	3 to 5	>5
Strength	Negative	Bare mention	Positive	Strong	Decisive

In order to compute $B_{10}$ for the hypotheses of interest of this work, it is necessary to define a sampling model for the confusion matrix $\varvec{Z}$ under each hypothesis. Notice that while evaluating the classification results, the total number of examples per class in the test set can be assumed as known. This assumption is usually known as *one margin fixed* and it means that the row marginals of $\varvec{Z}$ are known and then that the sampling model for each row of the confusion matrix is ${\mathrm{Bin}} (z_i|n_i,p_i)$, where $z_i$ is one of the two values of the $i$-th row (the other being $n_i - z_i$), $n_i$ is the known $i$-th row marginal and $p_i$ the unknown probability of predicting that class when the true class is $i$.

The sampling model under $H_0$ is that $p_1$ and $p_2$ are identical and drawn from one distribution, while, under $H_1,\, p_1$ and $p_2$ are drawn independently from their own distributions. The exact form of these distributions, called *prior distributions* $\pi (p_1,p_2|H_i)$, is a matter of debate and, in this work, we follow a recent result presented in [2] which is based on the use of a standard prior for $\pi (p_1,p_2|H_0)$ and the *intrinsic prior* [1] for $\pi (p_1,p_2|H_1)$. In this context, the idea of the intrinsic prior is that $\pi (p_1,p_2|H_1)$ should concentrate its mass around $\pi (p_1,p_2|H_0)$ to ensure that the inference process distinguishes between $H_0$ and close alternatives and gives less weight to extremely unlikely models. In [2], the degree of concentration is parametrised by the non-negative integer parameters $t_1$ and $t_2$, one for each row. From the definition of intrinsic prior and considering a uniform standard prior for $H_0$, i.e $\pi (p_1=p,p_2=p|H_0) = U_{(0,1)}(p)$, we get [2]:

7 $$\begin{aligned} \pi (p_1,p_2|t_1,t_2)=& \sum _{i=0}^{t_1} \sum _{j=0}^{t_2} \left( {\begin{array}{c}t_1\\ i\end{array}}\right) \left( {\begin{array}{c}t_2\\ j\end{array}}\right)\\&\times\, \frac{{\varGamma }(i + j + 1) {\varGamma }(t_1 + t_2 -i - j + 1)}{{\varGamma }(t_1 + t_2 + 2)} \\&\times\, {\mathrm{Beta}} (p_1| i + 1, t_1 - i + 1)\, {\mathrm{Beta}} (p_2 | j + 1, t_2 - j + 1) \end{aligned}$$

Then, the Bayes factor $B_{10}$ measuring the ratio of the evidence of $H_1$ against $H_0$ is

8 $$\begin{aligned} B_{10}(z_1,z_2,t_1,t_2)=& \frac{\int P(z_1,z_2|p_1,p_2,H_1) \pi (p_1,p_2|H_1) dp_1 dp_2}{\int P(z_1,z_2|p_1,p_2,H_0) \pi (p_1,p_2|H_0) dp_1 dp_2}  \\=& \left[ \frac{n_1+n_2+1}{(n_1+t_1+1)(n_2+t_2+1)}\right] \left[ \frac{(t_1+1)(t_2+1)}{t_1+t_2+1} \right]\\&\times\, {{n_1 + n_2} \atopwithdelims (){z_1+z_2}} \sum _{i=0}^{t_1}\sum _{j=0}^{t_2} \frac{{t_1 \atopwithdelims ()i}^2 {t_2 \atopwithdelims ()j}^2}{{{t_1+t_2} \atopwithdelims (){i+j}}{{n_1+t_1} \atopwithdelims (){z_1+i}}{{n_2+t_2} \atopwithdelims (){z_2+j}}}. \end{aligned}$$

The detailed derivation can be found in [2]. Notice that $B_{10}(z_1,z_2,t_1,t_2)$ is parametric in $t_1$ and $t_2$, which means that different degrees of concentration of $\pi (p_1,p_2|H_1)$ around $\pi (p_1,p_2|H_0)$ will lead to a different amount of evidence supporting $H_1$ against $H_0$. In [2], it is claimed that robust inference can be obtained only when $B_{10}(z_1,z_2,t_1,t_2)$ is stable w.r.t. variations of $t_1$ and $t_2$. Here we propose a conservative approach and define $B_{10}(z_1,z_2)=\min _{t_1,t_2 \in \{0 \ldots m\}} B_{10}(z_1,z_2,t_1,t_2)$. This means that when stability is an issue, we may not be able to claim that the classifier is able to discriminate among the classes.

In [2], it is shown how to extend Eq. 8 to the multi-class case, which we do not present here.

## Materials: the ADHD dataset

Our study refers to the ADHD-200 Initiative and dataset which is dedicated to support the scientific community in studying and understanding the neural basis of ADHD. The aim of the initiative is also meant to support the clinical community with the advance of objective tools for computer-aided diagnosis. Eight institutions collected neuroimaging datasets from almost one thousand young subjects (age 7–26) with and without ADHD. For each subject, multiple types of data were collected: phenotypic data, structural (T1) magnetic resonance imaging (MRI) data and functional MRI (fMRI) resting-state data. Accompanying phenotypic information included: age, gender, handedness and IQ measure. The ADHD-200 dataset is publicly available and freely distributed with the support of the International Neuroimaging Data-sharing Initiative.^1^

Even though the ADHD-200 dataset comprised three different levels of the ADHD disorder and the healthy controls, in this work, we restrict our analysis to the discrimination between two diagnostic categories, i.e. healthy controls and ADHD patients, by aggregating patients into one class. In the following, we refer to the whole dataset comprising the data of 1339 recordings from 923 subjects, where the diagnostic classes are distributed as follows: 62 % typically developing control and 38 % ADHD. For a few subjects, data were only partially available or corrupted. These subjects were excluded from our study.

In this work, we analyse the confusion matrices presented in [7]. We report a brief summary of the preprocessing and classification steps because a detailed presentation is beyond the scope of this paper and it can be found in [7]. The preprocessed data were retrieved from the NeuroBureau initiative^2^ and specifically from the Athena and Bruner pipelines managed by C. Craddock and C. Chu. Both structural (T1) volumes and statistical volume from fMRI resting-state recordings were transformed into vectors through the dissimilarity representation [9]. The classification algorithm adopted was the *extremely randomized tree* [4] with different cross-validation schemes.

In Sect. 4, we use the confusion matrices obtained in [7] from phenotypic data (denoted as PHEN) and fMRI resting-state data preprocessed according to the spatial multiple regression proposed in [10] (denoted as SMR0-9).

## Experiments

We compared the efficacy of the proposed test of independence against multiple standard metrics introduced in Sect. 2. Experiments were conducted on data from a simulated toy example and on real data concerning the computer-aided diagnosis of ADHD brain disease. The code of the experiments is freely available from https://github.com/FBK-NILab/brin2014.

### Simulated toy example

We expand the example introduced in Fig. 1 in order to clearly explain the differences between the proposed method and the standard metrics frequently used when evaluating classification results. In Table 2, we report the estimated PA, the Matthew correlation coefficient (MCC), the F1-score, the $\kappa$-statistic ($\kappa$) and Youden’s $J$-statistic ($J$) together with the proposed $\log (B_{10})$ for both the extreme cases introduced in Sect. 1 and two additional cases. The first of them refers to perfect prediction of all available examples and the second one to random prediction without considering the prior distribution of the diagnostic groups. In Table 3, we report the results from confusion matrices analogous to those in Table 2, but with exactly 1/5 of the examples. This second table aims at showing that only the proposed method is able to quantify the evidence in the data taking into account the test set size.

**Table 2:**
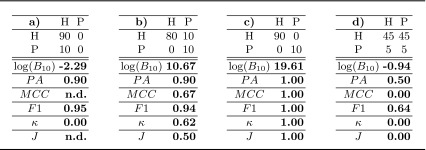
Confusion matrices on a test set of 100 examples, $\log (B_{10})$, prediction accuracy (*PA*), Matthews Correlation Coefficient (*MCC*), F1-Score (*F1*), $\kappa$-Statistic and Youden’s statistic (*J*) for different settings. (a) always predicting the dominant class, (b) errors only in the dominant class, (c) perfect prediction, (d) random prediction

**Table 3:**
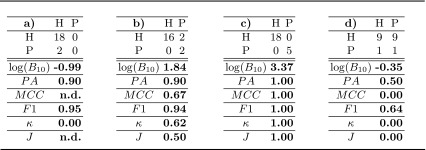
Confusion matrices on a test set of 20 examples obtained by dividing the values of the confusion matrices in Table 2 by a factor of 5. Accordingly, $\log (B_{10})$, prediction accuracy (*PA*), Matthews Correlation Coefficient (*MCC*), F1-Score (*F1*), $\kappa$-Statistic and Youden’s statistic (*J*) are reported

In Table 2, we observed that perfect prediction, i.e. (**c**), produces the highest scores for all the metrics considered, i.e. 1.0. The score of the proposed method, i.e. $\log (B_{10}) = 19.61$, means *decisive evidence* in favour of $H_1$, according to the interpretation guidelines in Table 1. So it agrees with all other metrics. The case of perfectly random prediction irrespective of the prior distribution, i.e. (**d**), is again correctly detected by all methods by scoring $0.0$, with the exception of the $F1$ score. The score of the proposed method, i.e. $\log (B_{10}) = -0.94$, is *negative* evidence for $H_1$ 3 in agreement with most of the standard metrics.

The comparison of the cases (**a**) and (**b**) of Table 2 shows that prediction accuracy ($PA$) and $F1$ score are not reliable for unbalanced datasets. The related confusion matrices represent opposite situations but those scores do not significantly change. For the confusion matrices in (**a**), the Matthews Correlation Coefficient (MCC) and Youden’s J score are undefined and only the $\kappa$-statistic correctly detects the difference between (**a**) and (**b**). In agreement with the $\kappa$-statistic, the proposed method reports *negative* evidence for $H_1$ for case (**a**) and *decisive* evidence for $H_0$ for case (**b**).^4^

In Table 3 the confusion matrices represent the same situations of those in Table 2 but with a reduced number of examples. This means that their interpretation in terms of scores must go in the same direction but the amount of evidence provided in Table 3 is much lower than that of Table 2 and the evaluation has to take that into account. In other words, we cannot draw the same conclusions from a test set of 20 examples with respect to a test set of 100 examples and this should be represented in the scores. As it can be seen from the comparison of the scores in Table 3 with respect to those in Table 2, all the standard metrics provide the same exact scores despite having $1/5$ of the data. Different from them, the proposed method shows a great reduction in value, correctly reflecting the reduced size of the test set. For example, in case (**b**), the amount of evidence in favour of $H_1$ is *decisive* in Table 2 ($\log B_{10} = 10.67$) but only worthy of a *bare mention* in Table 3 ($\log B_{10} = 1.84$).

### Real-data application

A standard 10-fold stratified cross-validation scheme was used to create the aggregated confusion matrices associated to the dataset described in Sect. 3. Table 4 presents them (SMR5, SMR7, SMR2 and PHEN) together with $\log (B_{10})$ of $H_1$ over $H_0$, estimated accuracy, and the other metrics introduced in Sect. 2. Each aggregated confusion matrix was computed as the sum of the confusion matrices of each cross-validation fold. The accuracies obtained for all single source dataset varied between 60 and 66 %. We focused on four cases that had a significantly different Bayes factor, but marginal differences in prediction accuracy.

**Table 4:**
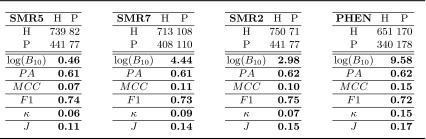
Confusion matrices and the related values of $\log (BF_{10})$ and of different standard metrics for ADHD classification. The confusion matrices considered here are from phenotypic data (PHEN) and from the spatial multiple regression of fMRI resting state (SMR2, SMR5, SMR7). See Sect. 3 and [7] for additional details. The standard metrics are the prediction accuracy ($PA$), Matthews correlation coefficient ($MCC$), F1-Score ($F1$), $\kappa$-Statistic and the Youden’s statistic ($J$)

The results shown in Table 4 about SMR5 and SMR7 have prediction accuracy of 61 % in both cases. The analysis of the confusion matrix by means of the test of independence reveals that SMR5 does not provide relevant information about ADHD diagnosis, while SMR7 provides *strong* evidence in support of $H_1$. The predictions in the latter case are therefore statistically dependent on the true class labels and a positive answer to the question, whether the classifier learned to discriminate the classes, can be given. Notice that $MCC,\, \kappa$ and $J$ show little increase from SMR5 to SMR7, making it difficult to detect such difference.

A substantially similar result can be obtained on SMR2 vs. PHEN, in Table 4. The prediction accuracy is again at the same level in both cases: while the SMR2 is found to obtain *positive* evidence, PHEN has a $\log (B_{10}) = 9.58$, which is *decisive* evidence for statistical dependence between predicted and true class labels. Other standard metrics, i.e. $MCC,\, \kappa$ and $J$, shows a small increase in value but the absence of interpretation guidelines, as those in Table 1, makes it difficult to understand the practical meaning of those changes.

Furthermore, the comparison of SMR7 vs. SMR2 shows another example for how the prediction accuracy may be misleading. The former has the lower prediction accuracy, but *strong* evidence ($\log (B_{10})=4.44$) that the classifier might have learned to discriminate the classes, while the latter has a slightly higher prediction accuracy, but only *positive* evidence ($\log (B_{10})=2.98$).

As a general comment, the ranking of relevance for diagnosis of the four different data sources is in agreement when considering the proposed method based on Bayesian inference and the Matthews correlation coefficient or the $\kappa$-statistic. The main difference is that the result of the proposed method has a direct interpretation in terms of evidence, while the significance of the differences in the values of the standard metrics across the confusion matrices remains to be determined.

## Discussion

In this work, we propose a novel method for the evaluation of classification results that overcome the limitations of commonly adopted metrics. The proposed method is based on the Bayesian inference framework and provides a measure of evidence in the data that can be easily interpreted by means of standard guidelines. This differs from standard metrics where guidelines for interpretation are not available due to the lack of a statistical foundation.

Additionally, in Sect. 4.1, we show that the proposed method agrees with standard metrics in many cases. But it is the only one able to provide the correct answer in more extreme cases, where standard metrics are either undefined or misleading.

In Sect. 4.2, on real data, we show that the proposed method distinguishes between data sources that are of importance for the discrimination between the diagnostic groups of ADHD from those who are not. This is sometimes in contrast with prediction accuracy that may lead to incorrect conclusions (see SMR7 vs SMR2).

The accurate detection of data sources which are irrelevant to diagnosis can lead to their exclusion from diagnosis protocols and therefore to improve the cost-benefit trade-off. The proposed Bayesian test of independence is an effective tool for such task.

## Footnotes

1 http://fcon_1000.projects.nitrc.org/indi/adhd200/

2
http://neurobureau.projects.nitrc.org/ADHD200/Data.html

3 Since $B_{01} = \frac{1}{B_{10}}$, this corresponds to *positive* evidence for $H_0$.

4 Those correspond to *positive* (**a**) and *negative* (**b**) evidence for $H_0$.
